# Safe liver resection following chemotherapy for colorectal metastases is a matter of timing

**DOI:** 10.1038/sj.bjc.6603670

**Published:** 2007-03-13

**Authors:** F K S Welsh, H S Tilney, P P Tekkis, T G John, M Rees

**Affiliations:** 1 Hepatobiliary Unit, North Hampshire Hospital, Basingstoke, UK; 2 Department of Biosurgery and Surgical Technology, St Mary's Hospital, Imperial College, London, UK

**Keywords:** colorectal metastases, liver resection, neoadjuvant chemotherapy

## Abstract

Neoadjuvant chemotherapy (NC) can improve the resectability of hepatic colorectal metastases (CRM). However, there is concern regarding its impact on operative risk. We reviewed 750 consecutive liver resections performed for CRM in a single unit (1996–2005) to evaluate whether NC affected morbidity and mortality. Redo hepatic resections or patients receiving adjuvant chemotherapy following primary resection were excluded. A total of 245 resections were performed in patients not requiring NC (control group) (mean age 63, 67% male) and 252 in patients who had NC (mean age 62, 67% male). The mean (s.d.) duration of surgery was less in the control group (241(64) *vs* 255(64)min, *P*=0.014) as was the mean blood loss (390(264) *vs* 449(424)ml, *P*=0.069). Postoperative mortality (2 *vs* 2%) and morbidity (27 *vs* 29%, *P*=0.34) was similar between groups. More NC patients developed septic (2.4%) or respiratory (10.3%) complications compared to controls (0 and 5.3%, *P*<0.03), with significantly more surgical complications if the interval between stopping NC and undergoing surgery was ⩽4 weeks (11%), compared to 5–8 (5.5%) or 9–12 (2.6%) weeks (*P*=0.009). The data suggest that liver resection for CRM is safe following NC. Early hepatobiliary involvement in multidisciplinary cancer care may lead to avoidance of potential perioperative adverse events.

Colorectal cancer is the third most common malignancy in the UK, with an incidence of over 34 000 (Cancer Research UK, 2006). Fifty per cent of these patients will either present with or develop liver metastases in the course of their disease (Scheele *et al*, 1990, 1995; Sugarbaker, 1990; Stangl *et al*, 1994). Of these, 20–30% are suitable for resection, which currently offers the greatest chance of cure, with a 5-year survival after curative liver resection being 25–40% (Hughes *et al*, 1986; Stangl *et al*, 1994; Scheele *et al*, 1995; Nordlinger *et al*, 1996; Rees *et al*, 1997; Fong *et al*, 1999; Adam *et al*, 2003).

Advances in surgical and anaesthetic technique have expanded the indications for liver resection in the past 20 years. However, in a landmark paper in 1996, Bismuth *et al* (1996) showed that neoadjuvant chemotherapy (NC) could downstage liver metastases. Their results demonstrated that 16% of previously inoperable patients subsequently underwent liver resection with curative intent and consequent 39% 5-year survival (Bismuth *et al*, 1996). This work, confirmed by others (Pozzo *et al*, 2004), has resulted in NC now being more widely used to improve the resectability of colorectal liver metastases. However, it is increasingly clear that chemotherapy damages the hepatic parenchyma, causing a chemotherapy-associated steatohepatitis (Fong and Bentrem, 2006), which may adversely affect recovery after hepatectomy (Elias *et al*, 1995; Pocard *et al*, 2001).

The aim of the study was to determine the impact of NC on postoperative morbidity and mortality following liver resection for colorectal metastases (CRM).

## PATIENTS AND METHODS

The records of 750 consecutive patients undergoing liver resections for CRM with a curative intent in a single institution between May 1997 and October 2005 were reviewed. Data were prospectively recorded on an Access® (Microsoft Corporation, Redmond, WA, USA) database consisting of 268 data fields compiled contemporaneously from a standardised proforma. Data validation was performed by requesting duplicate information from patient charts by a dedicated database officer. Details of the chemotherapy regimens prospectively recorded included drug types, duration of chemotherapy and the length of time (in weeks) between the cessation of chemotherapy and liver resection. The time period 1997–2005 was chosen to include all patients given NC before liver resection and a group of contemporaneous controls who had never been exposed to chemotherapy. Patients undergoing resections before May 1997 were excluded from the study to reduce potential bias from improved surgical and anaesthetic technique (*n*=179).

Those patients who had received neoadjuvant or adjuvant chemotherapy prior or following resection of the primary tumour (*n*=181) and patients undergoing redo hepatic resections (*n*=72) were excluded from the study. The remaining 497 patients were divided into two groups: a control group (*n*=245) who had surgery alone and a chemotherapy group (*n*=252) who received NC before liver resection.

The indications for NC have evolved throughout the time period of this study. Patients whose liver metastases were initially unresectable would be offered NC in the hope of downstaging their disease. Other potential indications for NC include patients with advanced primary tumours, those presenting with synchronous liver metastases, those with large or ill-located lesions close to either portal or hepatic venous structures, patients with multiple, bilobar metastases and patients with resectable extrahepatic metastases. Moreover, the current UK guidelines (Garden *et al*, 2006) recommend that patients with resectable liver metastases proceed straight to liver resection without NC. The definition of resectable disease remained constant throughout the study period. This was the ability to completely resect all liver metastases, regardless of size, number, distribution or width of resection margin, whereas preserving a sufficient volume of functioning hepatic parenchyma (usually 25–30% of functioning liver volume as estimated by computed tomography (CT) or magnetic resonance imaging), portal venous and hepatic arterial inflow, hepatic venous outflow and biliary drainage.

The anaesthetic and surgical techniques specific to hepatic resection have been described in detail previously (Rees *et al*, 1996). All patients underwent liver resection using low-CVP anaesthesia and intermittent inflow occlusion with the Pringle clamp to reduce blood loss during the procedure. The standard protocol used was repeated cycles of 20 min inflow occlusion and at least 5 min of reperfusion, with the minimum amount of inflow occlusion, ideally less than 60 min. The parenchymal transection was performed using the cavitron ultrasonic aspirator. Perioperative pain relief entailed thoracic epidural analgesia and, more recently, regional intermuscular bupivicaine infusion combined with patient controlled-analgesia (Basu *et al*, 2004).

The study end points included intraoperative parameters (duration of warm ischaemia and intraoperative blood loss) and postoperative mortality and morbidity. Postoperative mortality was defined as death during the same hospital admission or within 30 days of the operation date, if the patient was discharged earlier. Postoperative complications were documented prospectively and classified as minor, relevant (delayed patient discharge) or serious (complications requiring urgent medical or surgical intervention). The standard follow-up protocol included clinical examination, estimation of serum carcinoembryonic antigen level and abdominal ultrasonography or contrast-enhanced CT at 6 months, 1 year and annually thereafter until death.

### Statistical analysis

Continuous variables were compared using the analysis of variance and categorical variables were analysed using the *χ*^2^ test or Fisher's Exact test as appropriate. Trends over time were assesses using the *χ*^2^ test for trend (*γ*-correction). *P*-values of <0.05 were considered to represent statistical significance. Data were analysed using SPSS software (version 11.0) (SPSS Inc., Chicago, IL, USA).

## RESULTS

The patient demographics in the control and chemotherapy groups are detailed in Table 1. Although the two groups were matched for age and gender, there were significantly more patients with a clear reduction in World Health Organisation performance status in the chemotherapy group compared to the control group. In addition, patients who received NC were more likely to have presented with synchronous metastases and included a greater percentage of patients with Dukes' C primary disease. There was no difference in the number or size of liver metastases between groups. The details of the chemotherapy regimens in the chemotherapy group are shown in Table 2. Over half of the patients received oxaliplatin-based treatment. Table 3 shows the treatment intent of chemotherapy, classified as irresectable/downsized or resectable but given for other reasons, and the patients' radiological response, determined by CT scan.

The extent of liver resection performed was similar between the control and NC groups (Table 4), but whereas the Pringle clamp time was similar in both groups, the operating time was significantly longer for the chemotherapy patients (*P*=0.014) by a means of 14 min. In addition, the mean blood loss was higher in the chemotherapy group (449 ml) compared to the controls (390 ml), but this failed to reach statistical significance (*P*=0.067). Further examination of the largest subgroup of patients in the chemotherapy group, the 127 patients (50.4%) who received oxaliplatin-based chemotherapy was performed, with their operative details compared with those of the control patients. Again, there was no difference in Pringle clamp time (19.8 *vs* 17.8 min in the controls, *P*=0.12) and a significantly longer mean operating time (258 *vs* 241 min in the controls, *P*=0.012). However, in this subgroup analysis, a significantly greater mean (s.d.) blood loss in the patients receiving neoadjuvant oxaliplatin (473 (530) ml), compared to the control patients (390 (264) ml, *P*=0.046), was shown.

There were 10-postoperative deaths in the series (2%), five in each of the control and chemotherapy groups (*P*=1.0, Fisher's Exact test). The cause of death is detailed in Table 5. Similarly, there was no difference in overall morbidity, being 27% (66/245) in the control and 29% (73/252) in the chemotherapy group (*P*=0.34, Fisher's Exact test). The incidence of specific complications is shown in Table 6. There were significantly more complications from respiratory causes (*P*=0.027) or sepsis/abscesses (*P*=0.016) in the chemotherapy group. The six cases complicated by sepsis/abscess were two cases of sepsis/multiorgan failure, one epidural abscess, that led to paraplegia, one patient with MRSA cellulitis, one infected Hickman line and one patient who developed peritonitis of unknown cause. There was no difference in the incidence of hepatic insufficiency in the control (6.1%) or chemotherapy (7.5%) groups (Table 6). This was minor (i.e. did not delay discharge) in no patients in the control group and seven in the chemotherapy group, significant (i.e. delayed discharge beyond the 10th postoperative day) in 10 of the control and 10 of the chemotherapy patients and significant requiring intervention or admission to high-dependency or intensive therapy units in five control and two chemotherapy patients. All of the seven patients in this latter group underwent major hepatic resections. Of the five patients in the control group, four developed infective complications (three pneumonia, one small bowel perforation related to simultaneous closure of a loop ileostomy) and subsequent hepatic insufficiency as a result of this ‘second hit’ and one patient developed renal failure requiring haemofiltration, with hepatic insufficiency. Of the two patients in the chemotherapy group, one developed multiorgan failure including hepatic insufficiency following a right hepaticectomy and the other patient had hepatic insufficiency with infected ascites. One patient in each of the control and chemotherapy groups received MARS liver support therapy to treat their hepatic insufficiency (Steiner and Mitzner, 2002). One patient in each group died of multiorgan failure, on 19th (control patient) and 42nd postoperative day (chemotherapy patient).

**Figure 1:**
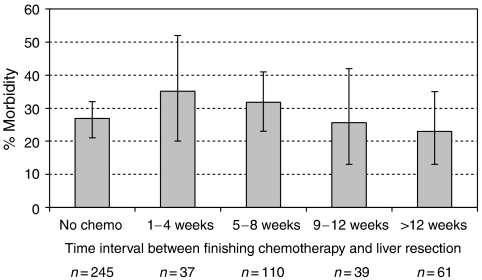
The effect of timing of cessation of chemotherapy on postoperative morbidity. ^*^P=0.96, *χ*^2^ test for trend.

**Figure 2:**
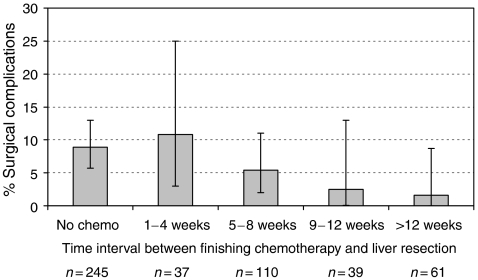
The effect of timing of cessation of chemotherapy on surgical complications. ^*^P=0.009, *χ*^2^ test for trend.

The time interval (in weeks) between finishing chemotherapy and undergoing surgery (Figure 1) did not impact significantly on postoperative morbidity (*P*=0.96, *χ*^2^ test for trend). However, Figure 2 illustrates the significant reduction in surgical complications with increasing time interval between the cessation of chemotherapy and hepatic resection (*P*=0.009).

**Figure 3:**
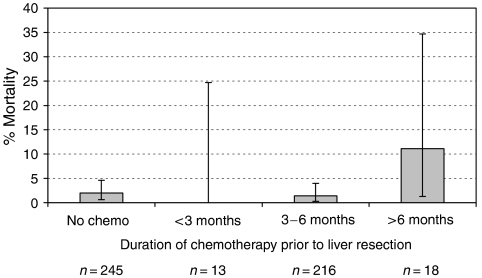
Effect of the duration of chemotherapy on postoperative mortality. ^*^P=0.65, *χ*^2^ test for trend.

The effect of the duration of chemotherapy on postoperative morbidity and mortality was then considered. Patients were grouped into those who had chemotherapy for less than 3 months, 3–6 months and more than 6 months (Table 7). Although there appears to be a trend towards a higher incidence of life-threatening and surgical complications if patients received more than 6 months of chemotherapy, this failed to reach statistical significance. Similarly, there was a striking increase in postoperative mortality (11%) in this group, but again this did not reach statistical significance (Figure 3) possibly reflecting an insufficient number of patients in this subgroup (*n*=18) to generate adequate statistical power.

## DISCUSSION

Neoadjuvant chemotherapy has become an integral part of the multidisciplinary management of metastatic colorectal cancer. It can result in the downstaging of disease and thus improve hepatic resection rates by 13–20% (Bismuth *et al*, 1996; Adam *et al*, 2001). It can also provide an *in vivo* assessment of tumour chemoresponsiveness, allowing clinicians to continue treatment for responders or change treatment for nonresponders. Furthermore, there is evidence that the response to chemotherapy is a prognostic indicator of survival even when subsequent liver resection is performed with curative intent (Allen *et al*, 2003; Adam *et al*, 2004). Lastly, for patients with synchronous disease or a short disease-free interval, NC allows treatment of liver metastases and systemic micrometastases simultaneously.

This study shows that NC before liver resection for CRM does not increase postoperative morbidity and mortality. This finding is supported by early data from the MD Anderson Cancer Centre on 108 patients who underwent major liver resection for CRM, of whom 61 had preoperative chemotherapy (27 5-FUFA and 34 5-FUFA+irinotecan) with no adverse effect on clinical outcome (Parikh *et al*, 2003). A similar study from Belghiti's group on 17 patients who had preoperative 5-FU, found no difference in postoperative morbidity compared to 18 patients who received no chemotherapy (Parc *et al*, 2000). These studies are in contrast to the preliminary results of the EORTC multicentre trial of preoperative chemotherapy (5-FUFA+oxaliplatin) and surgery for resectable colorectal liver metastases *vs* surgery alone (Nordlinger *et al*, 2005). Although the trial investigators also found no difference in 30-day mortality between the two groups, there was an increased incidence of postoperative complications in the chemotherapy arm (24.5%) compared to the surgery alone arm (13.3%). Transient liver failure (6.4 *vs* 1.6%), biliary fistula (5.5 *vs* 1.6%) and intra-abdominal infections (4.5 *vs* 0.8%) were all higher in those patients who had received NC compared to those undergoing surgery alone. Karoui *et al* (2006) have recently published a study examining the effects of preoperative chemotherapy on postoperative morbidity and mortality in 67 patients who underwent major hepatectomy under total vascular exclusion. These patients were particularly at risk of postoperative complications owing to the extent and complexity of the surgery. They showed that in this high-risk group, the 45 patients who had preoperative chemotherapy had significantly more postoperative complications (38%) compared with those who did not have chemotherapy (13.5%, *P*=0.03). Although the present results showed a higher incidence of hepatic insufficiency in those patients who had NC, this difference failed to reach statistical significance, possibly reflecting the low overall incidence of this complication in this series. A significant increase in respiratory and septic complications in the patients receiving NC, compared to the controls was, however, demonstrated.

There is some evidence that NC results in increased hepatic steatosis, which can impair postoperative liver regeneration and lead to hepatic insufficiency. The present study did not specifically assess the resected specimens for evidence of steatosis, unlike the MD Anderson study, which found an increased incidence of steatosis in those patients who had had preoperative chemotherapy, most marked in those who had received irinotecan. This is supported by work from Steve Strasberg's group, who evaluated liver biopsies of patients referred for resection of colorectal liver metastases. They found a significantly increased incidence of nonalcoholic steatohepatitis in those patients who had received neoadjuvant oxalipliatin or irinotecan, compared to patients who had 5-FU alone or those who did not have chemotherapy before liver resection (Fernandez *et al*, 2005). However, is there any evidence that steatosis affects clinical outcome? Pocard *et al* (2001) found that chemotherapy-induced steatosis impaired postoperative hepatic regeneration in patients undergoing hepatectomy for breast cancer metastases. Moreover, Didolkar *et al* (1989) showed that patients receiving preoperative chemotherapy had minimal liver regeneration on postoperative CT scanning and that these patients were more likely to develop postoperative hepatic insufficiency. A more recent paper from MD Anderson examined the histological changes associated with preoperative chemotherapy and correlated them with postoperative outcome after liver resection for CRM. Not only did they confirm their earlier findings that irinotecan was associated with a steatohepatitis, but they also found that patients with steatohepatitis had a significantly higher 90-day mortality (14.7%) compared to patients who did not have steatohepatitis (1.6%) (Vauthey *et al*, 2006). Others have investigated the effect of steatosis *per se* on clinical outcome following liver resection, finding that patients with steatosis had increased morbidity (Belghiti *et al*, 2000) and were more likely to have had preoperative chemotherapy and had more postoperative complications compared to controls with no steatosis (Kooby *et al*, 2003). A further histological study of the resected liver specimens following oxaliplatin-based NC has revealed a distinct pattern of liver injury. In 80% of the specimens, the authors found striking dilatation and rupture of the hepatic sinusoids, with progression to perisinusoidal and veno-occlusive fibrosis (Rubbia-Brandt *et al*, 2004). This finding of hepatic sinusoidal dilatation associated with oxaliplatin therapy has also been confirmed by others, but not proven to adversely affect outcome (Vauthey *et al*, 2006). It may however underlie our own clinical experience, and that of others, that the postchemotherapy liver parenchyma can be stiff and more liable to bleed during transection. This is reflected by our findings of significantly increased blood loss in those patients receiving oxaliplatin-based NC compared to the control patients and a significantly longer duration of surgery in all the chemotherapy patients compared to the controls.

The present data demonstrate how the postoperative morbidity, life-threatening complications and surgical complications were all greater in the small number of patients (*n*=18) who had chemotherapy for more than 6 months duration. These differences failed to reach statistical significance, possibly due to the small numbers involved (a Type II error). This is in contrast to an analysis of our early experience (1987–2002) where we found significantly increased morbidity in those patients who had had chemotherapy for more than 6 months (Basu *et al*, 2003). This led to our current practice of delaying surgery until the patient has recovered from any side effects associated with chemotherapy, such as bone marrow suppression and when their general fitness is optimised. Despite this patient-centred approach, the performance status in the chemotherapy patients was still significantly reduced compared to the controls, before liver resection. There is little published evidence regarding the impact of dose or duration of preoperative chemotherapy on postoperative morbidity following liver resection for CRM. One study from Germany on 30 patients who underwent either three or six cycles of preoperative oxaliplatin-based chemotherapy (FOLFOX regimen) found no difference in postoperative outcome between the two regimens (Lorenz *et al*, 2003). Indeed, in (Bismuth *et al*, 1996) landmark paper on downstaging chemotherapy (using an oxaliplatin-based regimen), the mean duration of chemotherapy needed to confer operability was approximately 8 months (Bismuth *et al*, 1996) and yet the consequent morbidity (26%) and mortality (zero) were low. However, the recent paper from Nordlinger's group, examining high-risk patients undergoing major hepatectomy under total vascular exclusion, reported a positive correlation between postoperative morbidity and the number of cycles of preoperative chemotherapy (Karoui *et al*, 2006).

In this study, hepatic resection was performed in 12 patients who had had a complete response to chemotherapy. The authors believe it is important to resect all documented sites of disease within the liver, even if some lesions have shown a complete response to chemotherapy, as there is evidence that these metastases will eventually recur. Benoist *et al* (2006) prospectively followed 38 patients who had multiple colorectal liver metastases and who received NC before hepatic resection. They found that 55 out of 66 metastases (83%) that had shown a complete radiological response to chemotherapy had either residual (macroscopic or microscopic) disease at operation, or recurrent disease at a year.

In conclusion, this is the largest study to date, which has addressed the question as to whether NC increases the risk of liver resection for CRM. The present study confirmed that chemotherapy before resection of colorectal liver metastases is safe, despite the performance status of the patients receiving chemotherapy being lower than that of the controls, and the surgery taking longer, with more associated blood loss. However, whereas NC has become an integral part of the quest to improve resection rates and survival, no treatment is without risk, as highlighted by the increased respiratory and septic complications in the chemotherapy group. The present study suggests that avoiding giving chemotherapy for a prolonged duration and carefully timing subsequent liver surgery to each individual patient's recovery from chemotherapy may result in immediate outcomes comparable to those patients who have not received chemotherapy. We would advocate the involvement of hepatobiliary surgeons at an early stage in the multidisciplinary management of these patients, allowing appropriate planning of surgical and chemotherapeutic strategies.

## Figures and Tables

**Table 1:** Patients' demographic characteristics of patients undergoing hepatic resection for colorectal liver metastasis with and without neo-adjuvant chemotherapy; control group *vs* chemotherapy group

	**Control (*n*=245)**	**Chemotherapy (*n*=252)**	***P*-value**
Age at liver surgery; mean (s.d.) years	63 (10.8)	62 (9.8)	0.098^a^
Male gender	163 (67%)	168 (67%)	1.0^b^
			
*Performance status; n* (%)			0.028^b^
Normal/minimal reduction	166 (67.8%)	149 (59.1%)	
Clear reduction	79 (32.2%)	103 (40.9%)	
			
*Duke's Stage of primary tumour; n* (%)			<0.001^b^
Dukes' A or B	145 (59.2%)	79 (31.3%)	
Dukes' C	99 (40.4%)	167 (66.3%)	
			
*Timing of liver metastases; n* (%)			<0.001^b^
Synchronous	95 (38.8%)	151 (59.9%)	
Metachronous	150 (61.2%)	101 (40.1%)	
			
*Number of liver metastases; n* (%)			0.26^b^
1–3	211 (86.1%)	207 (82.1%)	
>3	33 (13.5%)	43 (17.1%)	
			
*Size of liver metastases; n* (%)			0.64^b^
<5 cm	164 (66.9%)	173 (68.2%)	
>5 cm	81 (33.1%)	78 (31.0%)	

s.d.=standard deviation.

a ANOVA.

b Fisher's Exact test.

**Table 2:** Chemotherapy agents

**Drug(s)**	**Number of resections (%)**
Oxaliplatin+5-FUFA	127 (50.4)
5-FUFA or Capcitabine alone	55 (21.8)
Irinotecan±5-FUFA	17 (6.7)
Mitomycin C+5-FUFA	9 (3.6)
Other	34 (13.5)
Not recorded	10 (4.0)

5-FUFA=5-flurouracil/ folinic acid.

**Table 3:** Treatment intent of and radiological (CT) response to neoadjuvant chemotherapy

**Response to neoadjuvant chemotherapy**	**Resectable (*n*=161)**	**Inoperable, down sized (*n*=91)**
No response/ stable disease (*n*)	59	3
Partial response (*n*)	91	87
Complete response (*n*)	11	1

**Table 4:** Operative details

	**Control (*n*=245)**	**Chemotherapy (*n*=252)**	***P*-value**
Major resections (⩾3 segments); *n* (%)	144 (58.8)	153 (60.7)	0.36^a^
Duration of surgery (min); mean (s.d.)	241.3 (64.2)	255.4 (63.6)	0.014^b^
Duration pringle clamp (min); mean (s.d.)	17.8 (23.4)	21.4 (25.9)	0.19^b^
Blood loss (ml); mean (s.d.)	390.4 (263.8)	448.8 (423.5)	0.067^b^

s.d.=standard deviation.

a Fisher's Exact test.

b ANOVA.

**Table 5:** Cause of death in patients undergoing hepatic resection for colorectal liver metastasis with (*n*=252) and without (*n*=245) neo-adjuvant chemotherapy

**Patient**	**Cause of death**
*Control group*	
1	Chest Infection
2	Collapse at home – cause of death unknown
3	Sepsis/multiorgan failure
4	Multiorgan failure
5	Ischaemic colitis/haemorrhage/renal failure
	
*Chemotherapy group*	
1	Sepsis/multiorgan failure
2	Peritonitis, cause unknown
3	Pneumonia/ARDS
4	Cardiac arrest 3rd postoperative day
5	Sepsis/multiorgan failure

ARDS=acute respiratory distress syndrome.

**Table 6:** Specific postoperative complications in patients undergoing hepatic resection for colorectal liver metastasis with (*n*=252) and without (*n*=245) neoadjuvant chemotherapy

	**Control *n* (%)**	**Chemotherapy *n* (%)**	***P*-value^a^**
Abscess/sepsis	0	6 (2.4)	0.016
Bile leak	3 (1.2)	2 (0.8)	0.487
Bleed	3 (1.2)	1 (0.4)	0.30
Cardiac	12 (4.9)	6 (2.4)	0.10
DVT/PE	3 (1.2)	7 (2.8)	0.18
Hepatic insufficiency	15 (6.1)	19 (7.5)	0.33
Respiratory	13 (5.3)	26 (10.3)	0.027
UTI	8 (3.3)	4 (1.6)	0.178
Wound infection	7 (2.9)	2 (0.8)	0.081

DVT=deep vein thrombosis; PE=pulmonary embolus; UTI=urinary tract infection.

a Fisher's Exact test.

**Table 7:** Effect of the duration of chemotherapy on morbidity and mortality *n*=(%) following hepatic resection for colorectal liver metastasis

		**Duration of chemotherapy**			
	**Control**	**<3 m**	**3–6 m**	**>6 m**	
**Outcome**	***n*=245**	***n*=13**	***n*=216**	***n*=18**	***P*-value** ^*^
Any morbidity	66 (27%)	4 (31%)	61 (28%)	7 (39%)	0.487
Life-threatening complications	13 (5%)	0 (0%)	11 (5%)	3 (17%)	0.438
Medical complications	53 (22%)	4 (31%)	55 (26%)	4 (22%)	0.373
Surgical complications	22 (9%)	0 (0%)	9 (4%)	3 (17%)	0.230
Mortality	5 (2%)	0 (0%)	3 (1%)	2 (11%)	0.645

*χ*^2^ test for trend.

In *n*=5 (2%) patients the duration of chemotherapy was not recorded.
